# Genetic Linkage Map of a High Yielding FELDA Deli×Yangambi Oil Palm Cross

**DOI:** 10.1371/journal.pone.0026593

**Published:** 2011-11-01

**Authors:** Tzer-Ying Seng, Siti Hawa Mohamed Saad, Cheuk-Weng Chin, Ngoot-Chin Ting, Rajinder Singh Harminder Singh, Faridah Qamaruz Zaman, Soon-Guan Tan, Sharifah Shahrul Rabiah Syed Alwee

**Affiliations:** 1 Federal Land Development Authority Malaysia Biotechnology Centre, Federal Land Development Authority Malaysia Agricultural Services Sdn. Bhd., Kuala Lumpur, Malaysia; 2 Advanced Biotechnology and Breeding Centre, Malaysian Palm Oil Board, Kajang, Malaysia; 3 Institute of Bioscience, Universiti Putra Malaysia, Serdang, Malaysia; 4 Department of Cell and Molecular Biology, Universiti Putra Malaysia, Serdang, Malaysia; University of California, United States of America

## Abstract

Enroute to mapping QTLs for yield components in oil palm, we constructed the linkage map of a FELDA high yielding oil palm (*Elaeis guineensis*), hybrid cross. The parents of the mapping population are a Deli dura and a pisifera of Yangambi origin. The cross out-yielded the average by 8–21% in four trials all of which yielded comparably to the best current commercial planting materials. The higher yield derived from a higher fruit oil content. SSR markers in the public domain - from CIRAD and MPOB, as well as some developed in FELDA - were used for the mapping, augmented by locally-designed AFLP markers. The female parent linkage map comprised 317 marker loci and the male parent map 331 loci, both in 16 linkage groups each. The number of markers per group ranged from 8–47 in the former and 12–40 in the latter. The integrated map was 2,247.5 cM long and included 479 markers and 168 anchor points. The number of markers per linkage group was 15–57, the average being 29, and the average map density 4.7 cM. The linkage groups ranged in length from 77.5 cM to 223.7 cM, with an average of 137 cM. The map is currently being validated against a closely related population and also being expanded to include yield related QTLs.

## Introduction

The oil palm, *Elaeis guineensis* Jacq., is the world's most productive oil crop. It is only grown in a belt stradding the equator, usually from 10°N to 10°S, in smallholdings to large plantations, and the world's biggest producer, Federal Land Development Authority Malaysia (FELDA), is a unique combination of both. A key contributor to FELDA's production, in such diverse socio-agro environments, is quality seeds from its large breeding programme. It is the premier oil palm seed supplier in Malaysia where about 40% of world palm oil is produced.

To accelerate its breeding progress, FELDA is using DNA markers, as oil palm breeding is time consuming and costly due to the long generation cycles, large plant size and an evaluation period needed of 10–15 years. The first genetic linkage map of the palm, based on RFLP markers and a *tenera*×*tenera* cross as the mapping population, was published in 1997 [1]. *Tenera* refers to an oil palm type which nuts have thin shells, and hence more oil-bearing pulp, compared to the naturally more common *dura* type which has thick-shell nuts. A shell-less form, the *pisifera*, exists but is female sterile. Shell thickness is influenced by a single gene and the *tenera* type results from a cross between *dura* and *pisifera*. The commercially-cultivated *tenera* is produced as a F_1_ hybrid between inbred *duras* and *pisiferas*. Not surprisingly in [1] above and later work the preference was for populations that segregated for the shell gene in the parallel search for a maker closely linked to this economically important trait. The choice of markers was influenced by developments in marker systems. Hence the above was followed by mapping a *tenera*×*pisifera* cross with RAPD markers while seeking a marker for the shell trait through bulk segregation [2]. In 2001, the first quantitative trait loci (QTLs) for yield on the same population as Mayes *et al.* [1] were mapped [3]. Among the outputs of the multi-institutional EU Link2Palm project (2001–4) was publication of the first dense oil palm genetic linkage map involving a large number of SSR and AFLP markers [4]. The same mapping population (LM2T×DA10D) was cited in the map published in 2005 [5]. In the last five years, researchers at Malaysian Palm Oil Board (MPOB) have used markers for germplasm diversity analysis [6]–[9], linkage to monogenic traits of fruit colour and shell thickness, map construction and QTLs for yield and fatty acid composition of the oil [8], [10]–[17].

We report here the construction of a linkage map of a FELDA high-yielding *dura*×*pisifera* cross using SSR markers posted by CIRAD in the public domain, from MPOB and from those developed at FELDA. Additional primer combinations were designed to produce AFLP markers for map saturation [18]–[20].

## Materials and Methods

### Mapping population

The mapping population is a high-yielding *dura*×*pisifera* cross, coded DA41, planted at FELDA's main research station in Jerantut, Malaysia. The female parent (ARK86D) is a *dura*, from selfing and sib-mating in small populations over seven generations from a few founder palms of Deli, Sumatra origin. The male parent (ML161P) is a *pisifera* descended through sib-mating in very small populations over four generations, beginning from selections at Yangambi in the Congo. Both parents are extensively used in FELDA's breeding programme, extant and have unambiguous pedigree information as well as productivity and growth data of themselves and their progenies. While the cross is high yielding there is notable segregation for the components of the high yield. In the breeding trial the cross is represented by 562 palms of which 120, with full growth and productivity records, were selected for the mapping work reported here.

### Genomic DNA extraction

Genomic DNA was extracted from mature leaflets (from frond 17, the youngest fully-opened frond being #1. Frond 17 is about 8.5 months after Frond 1, and about the middle frond in the palm canopy) of both parents and individuals of the mapping population using the modified cetyltrimethylammonium bromide (CTAB) method, suitable for stored mature oil palm leaf [21]. DNA quality was ascertained through gel electrophoresis on 0.8% agarose gel while the DNA quantity was estimated using a NanoDrop® ND-1000 spectrophotometer (NanoDrop Technologies Inc).

### AFLP Analysis

AFLP analysis was performed with some modifications [22] - the restriction and ligation done in a single reaction. Genomic DNA (90 ng) was double digested with *Eco*RI and *Mse*I enzymes (New England Biolabs) and the mixture incubated at 37°C for 2 hours. In the next step, the reaction mixture was diluted with 189 µl TE_0.1_ buffer. Two primers, used for PCR amplification, were designed based on the adaptor sequences and restriction site sequences. Selective nucleotide sequences were added to the 3′ end of each primer. PCR amplification was conducted in two steps: preselective and selective. For pre-amplification, the *Eco*RI primer (5′-GACTGCGTACCAATTC A-3′) had an adenine (A) and the *Mse*I primer (5′-GATGAGTCCTGAGTAA C-3′) a cytosine (C) as additional base at the 3′-end. The *Eco*RI and *Mse*I primers in the selective amplification used three additional nucleotides at the 3′ end; therefore, each primer combination amplified different subsets of all the fragments in the total digest. Pre-amplification PCR was done in a thermal cycler programmed to 72°C for 2 min; 20 cycles of denaturing for 20 s at 94°C, annealing for 30 s at 56°C, and extending for 2 min at 72°C followed by final extension for 30 min at 60°C. The pre-amplified DNA was diluted 1∶19 with TE_0.1_ buffer. A volume of 1.5 µl of the diluted product from pre-selective amplification was used for selective amplification in a reaction tube containing 8.5 µl selective amplification mixtures. The reaction mixture was transferred to a thermal cycler pre-heated to 94°C and the DNA amplified in ten cycles of: 20 s at 94°C, 30 s at 66°C (decrease 1°C every cycle) and 2 min at 72°C, followed by a further 20 cycles with a lower annealing temperature of 56°C. The final 60°C extension step was extended for 30 min. The PCR products of selective amplifications were separated by capillary electrophoresis on an ABI 3130xl Genetic Analyzer (Applied Biosystems, USA), and detected by fluorescence as the *Eco*RI site-specific primers were labeled with blue (6FAM™) or green (HEX™) fluorescent dyes. An internal standard, GeneScan™ 500 LIZ™, labelled with a red (ROX) dye was used for size calling, to allow co-loading of three reactions. For selective amplification, a total 80 primer combinations (PCs, *EcoR*I/*Mse*I) were tested on both parents and ten randomly picked progeny individuals of cross DA41. The amplification products were evaluated using GeneMapper® Software v4.0. to analyze data from samples loaded and run on the ABI 3130xl Genetic Analyser. The 30 most informative, in terms of number of polymorphic fragments detected, clear dominance inheritance patterns and reproducibility, were used for linkage analysis and mapping.

### Microsatellite analyses

Oil palm SSR primers isolated by FELDA (unpublished), MPOB (unpublished, except some) and CIRAD (http://tropgenedb.cirad.fr/oilpalm/publications.html.) were used, the last synthesized locally based on the published sequences. The combined total of 800 primers were tested on both parents and ten individuals of the cross DA41 as in the AFLP primer tests. From the 800, 247 (30.8%) that generated robust and easily interpretable genotypes were selected for linkage analysis and mapping. The remaining 553 primer pairs either did not amplify or amplified complex patterns of segregation and were not studied further. The 247 informative primer pairs were used to screen the entire mapping population. The PCR reaction was done in 15 µl mixture containing 0.075 U *Taq* Polymerase (INVITROGEN, BRAZIL), 10× PCR Buffer, 3 mM MgCl_2_, 0.3 mM dNTPs and 2 µM of each primer. The PCR was performed in thermal cyclers with initial denaturation for 60 s at 95°C; 35 cycles denaturation of 30 s at 94°C, annealing for 60 s at 52°C and extending for 120 s at 72°C followed by final extension for 15 min at 72°C.

SSR assays of selected primer pairs were performed using automated infrared fluorescence with a Li-Cor IR2 4200 sequencer (LI-COR, Lincoln, Nebraska, USA) [23]. For every forward SSR primer, a 5′tail was added with an M13 sequence 5′-GGA AAC AGC TAT GAC CAT-3′ [24]-[25] which permitted concurrent fluorescence labeling of PCR products by a third primer (M13) with an incorporated Infrared dye (IR700 or IR800) together with the reverse primer [26]. The PCR was performed in a 15 µl reaction mixture containing 50 ng DNA, 1× PCR buffer (−MgCl_2_), 0.2 mM dNTP mix, 0.2 U Taq Polymerase (INVITROGEN, BRAZIL), 2.0 mM MgCl_2_, 0.2 µg/µL BSA, 1 µl of three Primer Mixes (5 µM M13-tailed forward primer, 5 µM Untailed reverse primer and 0.25 µM IRD labeled-M13 primer) and sterile deionized water to make up to 15 µl. Following an initial denaturation step of 1 min at 95°C, also to heat activate the DNA polymerase, PCR was performed over 35 cycles at 94°C for 30 s, 52°C for 60 s and 72°C for 120 s and a final elongation step at 72°C for 15 mins. IR700- or IR800-labeled PCR products were separated using 6.5% polyacrylamide gel electrophoresis and sized by the IR fluorescence scanner of the sequencer.

### PCR–RFLP analysis

A total of 41 EST-RFLPs with at least one marker per linkage group were selected from the MPOB oil palm linkage maps [17], their EST sequences obtained and restriction enzyme (RE) cutting sites ascertained. The restriction sites for 21 REs (*Afa*I, *Alu*I, *Bam*HI, *Bcl*I, *Bgl*II, *Bst*NI, *Dra*I, *Eco*RI, *Hae*III, *Hind*III, *Hpa*II, *Mse*I, *Not*I, *Pst*I, *Sma*I, *Sst*I, *Taq*I, *RsaI*, *Hinc*II, *Msp*I and *Xba*I) were detected using BioEdit version 7.0.5.2 [27]. Primer pairs covering the restriction sites were designed using Primer 3 [28].

Amplification of the amplicon was carried out in the PCR reaction mix of 22 µl 10× PCR buffer (NEB, USA), 4.4 mM dNTPs, 5 µM forward primer, 5 uM reverse primer, 3.5 U *Taq* DNA polymerase (NEB, USA) and 100 ng template DNA. PCR was performed in a Perkin Elmer 9600 thermocycler as follows: denaturation at 95°C for 3 min; 40 cycles of 95°C for 30 s, annealing (the temperature depending on the primer) for 30 s and 72°C for 1 min, and a final extension at 72°C for 20 min. The PCR products (pre-RE digestion) were checked on 2.0% agarose electrophoresis in 1× TBE buffer at 100 V for 2 hours. Only the well amplified products were digested with REs and fragmented on 3.0% agarose in TBE buffer at 100 V for 2 hrs. A similar process was used for genotyping the detected polymorphic primer-pairs on the entire mapping population.

The PCR–RFLPSs were screened for polymorphism on the same panel used in the AFLP and SSR screening. The six primers, SFB00154_*Mse*I, SFB00221_*Mse*I, MET00004_*Taq*I, SFB00020_*Rsa*I, CB00055_*Alu*I and CA00026B_*Hinc*II, with five enzyme combinations found to be polymorphic were used to genotype the 120 progenies and two parents of cross DA41.

### Data Analysis

Polymorphic DNA fragments were scored as present/absent in parents and progenies. The genotype configurations of the SSR, AFLP and PCR–RFLPS markers segregating in the mapping population were identified and coded following the nomenclature [29] and diagramme [30] for a cross between two heterozygous parents. Chi-square tests, at thresholds of P≤0.05 and P≤0.01, for segregation distortion for all locus situations, comparing the observed and expected ratios for each possible locus configuration (1∶1, 3∶1, 1∶1∶1∶1 or 1∶2∶1) were performed. AFLP markers showing skewed segregation ratios at P≤0.01 were excluded as we considered the skew to be due to identical or very close electrophoretic mobilities of non-homologous fragments [22]. AFLP or SSR loci with 100% similarity were discarded to simplify the computation of locus order.

### Construction of Genetic Linkage Map

DA41 is a cross of two heterozygous parents and hence treated as a “double-pseudo-test cross” [31]. As such, the segregations of marker loci in this population were considered to be like those in a F2 population. First, parental linkage groups were constructed based on the markers/fragments specific to each parent. This was followed by taking fragments common to both parents as anchor points and integrating them into linkage groups [29], [32]. These anchor markers were linked with zero or small recombination frequencies (<3 cM) with individual fragments from both parents. Then, linked fragments were arranged into linkage groups using a minimum, commonly accepted LOD threshold of 3.0 between consecutive markers. MAPRF7 was used to perform the required linkage analysis between marker fragments, estimation of recombination frequencies, determination of linear order between linked loci including multipoint linkage analysis and expectation-maximization (EM) algorithm for handling missing data [29], [33].

## Results

### Generation of polymorphic DNA markers

Each of the 247 informative SSR primer pairs was screened for polymorphism between its female Deli dura (ARK86D) and male Yangambi pisifera (ML161P) parents. All the primers, except 15, amplified a single locus each, resulting in an overall average of 1.06 markers per probe. One of the resulting 263 markers produced a distorted segregation ratio in the progeny and was excluded from further analysis (Table 1). Of the remaining 262, 99 (37.6%) were fully informative, i.e. segregated in both parents with three or four different alleles each, 162 testcross and 99 intercross markers. In the first group, 60 (37%) were heterozygous in the female parent (ARK86D) and the rest (102, or 63%) heterozygous in ML161P, the male parent. The SSR allele segregation patterns, excluding the 3∶1 segregation class which was not scored, fell into four of the nine allelic configuration classes [29] for a cross between heterozygotes with up to four alleles a locus (Table 2).

**Table 1:** Characteristics of AFLP, PCR–RFLP and SSR markers screened in DA41 mapping population.

Marker type	No. probes/primer pairs tested	No. informative probes/primer pairs	No. polymorphic loci identified	No. markers showing 3∶1 segregation	No. markers showing 1∶1 segregation	No. markers showing distorted segregation
AFLP	80	30	402	92	307	66
PCR-RFLP	22	6	6	0	6	0
SSR	800	247	263	5	258	1
Total	902	283	671	97	571	67

**Table 2:** Segregation patterns of progeny phenotypes for markers in DA41 mapping population.

Number alleles	Segregation pattern	Female parent (ARK86D)			Male parent (ML161P)			Total
		AFLP	PCR-RFLP	SSR	AFLP	PCR–RFLP	SSR	
1–2	1∶1	175	2	60	132	2	102	473
2	1∶2∶1	-	2	10	-	2	10	12
3	1∶1∶1∶1	-	-	45	-	-	45	45
4	1∶1∶1∶1	-	-	44	-	-	44	44

From the 80 AFLP PCs tested, 30 were informative and generated 402 polymorphic markers of which 58% were heterozygous in the female parent, ARK86D, and absent in the male parent, and the rest heterozygous in the latter. The number of polymorphic markers per PC ranged from 2 to 25, with a mean of 13.4. The markers heterozygous in both parents (intercross markers), being less informative co-migrating AFLPs (3∶1 segregation ratio), were discarded - despite their theoretical ability to align genetic maps [34] - as their linkage phase was not ascertained.

Of the 41 PCR–RFLPs primers tested, 32 amplified a single band, 3 amplified 2 bands, 4 more than 2 bands while two failed to amplify any over several optimization attempts. Only the primers that amplified 1–2 bands were continued with restriction enzyme digestion. Of them, 6 in combination with five enzymes revealed polymorphism and these 6 informative primers were used to genotype the entire mapping family. Of the six, two each were dominant loci from the parents, ARK86D and ML161P, and the remaining two common and co-dominant from both parents.

### Linkage Analysis and Maps

**Figure 1:**
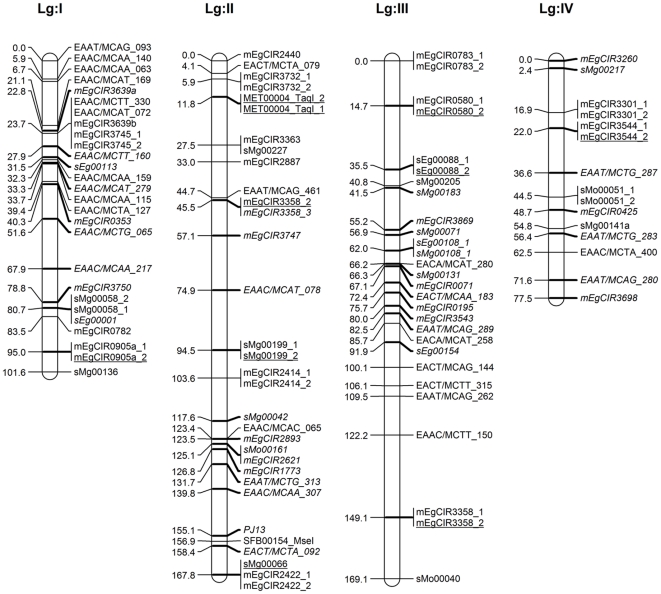
Integrated linkage map of FELDA's oil palm cross DA41 (ARK 86×ML 161) using MAPRF7 programme with Kosambi mapping function (Linkage Group I–IV). Marker names are shown to the right of each LG, with map distances (in cM) to the left. The map consists of 479 marker loci (331 SSRs, 142 AFLPs and 6 PCR–RFLPs) with 168 anchor points. Markers indicated in normal front are from map ARK86 while markers in italics are from map ML161, and markers representing an anchor point are underlined. Marker types and designations are as follows: SSRs (CNI, DHP, mEgCIR, PJ, sEg, sMg and sMo); RFLPs (CA, CB, MET and SFB); AFLPs (EAAC, EAAT, EACA, EACC, EACT, EAGA and EAGG).

**Figure 2:**
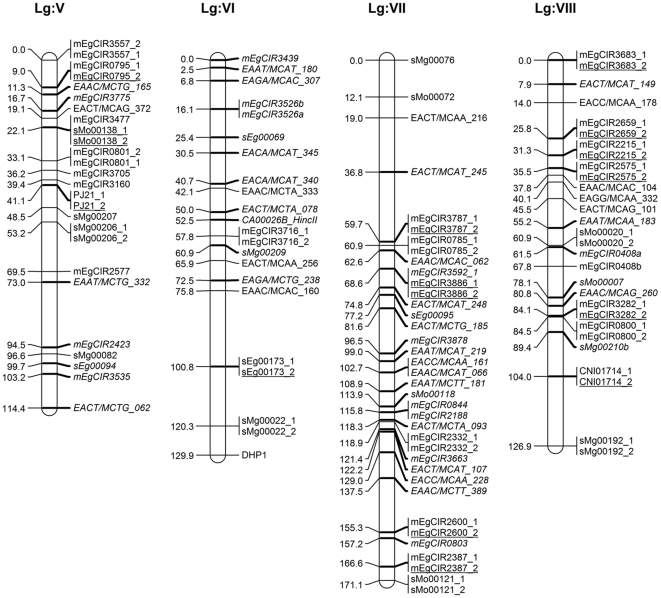
Integrated linkage map of FELDA's oil palm cross DA41 (ARK 86×ML 161) using MAPRF7 programme with Kosambi mapping function (Linkage Group V–VIII). Marker names are shown to the right of each LG, with map distances (in cM) to the left. The map consists of 479 marker loci (331 SSRs, 142 AFLPs and 6 PCR–RFLPs) with 168 anchor points. Markers indicated in normal front are from map ARK86 while markers in italics are from map ML161, and markers representing an anchor point are underlined. Marker types and designations are as follows: SSRs (CNI, DHP, mEgCIR, PJ, sEg, sMg and sMo); RFLPs (CA, CB, MET and SFB); AFLPs (EAAC, EAAT, EACA, EACC, EACT, EAGA and EAGG).

**Figure 3:**
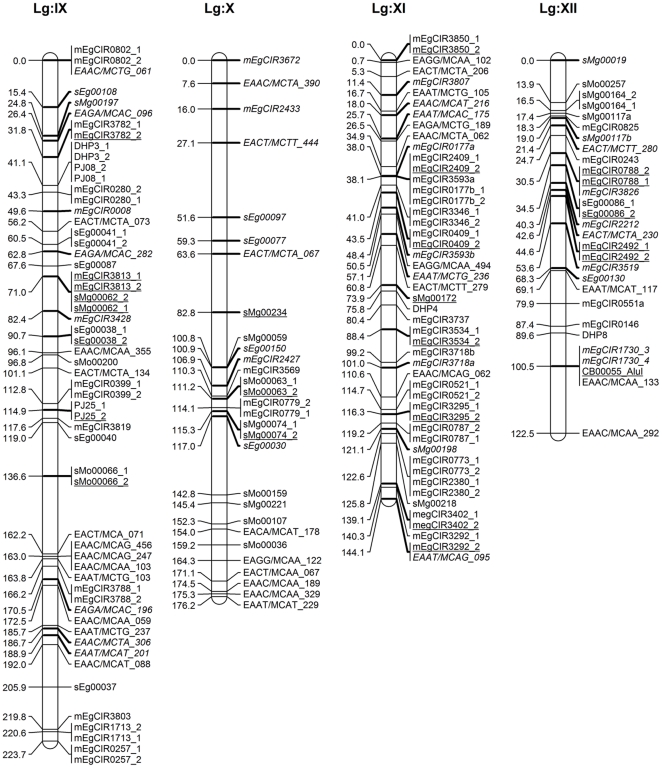
Integrated linkage map of FELDA's oil palm cross DA41 (ARK 86×ML 161) using MAPRF7 programme with Kosambi mapping function (Linkage Group IX–XII). Marker names are shown to the right of each LG, with map distances (in cM) to the left. The map consists of 479 marker loci (331 SSRs, 142 AFLPs and 6 PCR–RFLPs) with 168 anchor points. Markers indicated in normal front are from map ARK86 while markers in italics are from map ML161, and markers representing an anchor point are underlined. Marker types and designations are as follows: SSRs (CNI, DHP, mEgCIR, PJ, sEg, sMg and sMo); RFLPs (CA, CB, MET and SFB); AFLPs (EAAC, EAAT, EACA, EACC, EACT, EAGA and EAGG).

**Figure 4:**
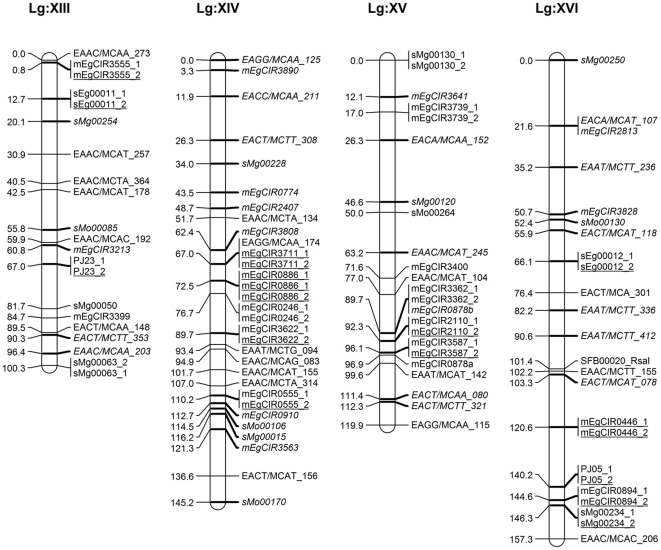
Integrated linkage map of FELDA's oil palm cross DA41 (ARK 86×ML 161) using MAPRF7 programme with Kosambi mapping function (Linkage Group XIII–XVI). Marker names are shown to the right of each LG, with map distances (in cM) to the left. The map consists of 479 marker loci (331 SSRs, 142 AFLPs and 6 PCR–RFLPs) with 168 anchor points. Markers indicated in normal front are from map ARK86 while markers in italics are from map ML161, and markers representing an anchor point are underlined. Marker types and designations are as follows: SSRs (CNI, DHP, mEgCIR, PJ, sEg, sMg and sMo); RFLPs (CA, CB, MET and SFB); AFLPs (EAAC, EAAT, EACA, EACC, EACT, EAGA and EAGG).

A total 294 segregating markers (258 SSRs, 30 AFLPs and 6 PCR–RFLPs) contributed 805 marker loci (490 SSRs, 307 AFLPs and 8 PCR-RFLPs) were used for linkage analysis and to construct maps of each parent separately and in an integrated map. The linkage group characteristics are summarised in Tables 3 and 4, and integrated (DA41) maps displayed in Figures 1, 2, 3 and 4. Following convention, the marker names are given on the right and their positions in cM [35] on the left of each marker bar on each linkage group. On the integrated map, the markers common to both parents, most of which represent anchor points, are underscored while those from parent 1 are shown in italics.

**Table 3:** Characteristics of genetic linkage groups of parents of mapping population DA41^1^.

Linkage group	Parent 1 (ARK86)					Parent 2 (ML161)				
	IP1	CM	TM	cM	AMD	IP2	CM	TM	cM	AMD
1	17	2	19	80.2	4.2	15	1	16	100.2	6.3
2	15	4	19	157.6	8.3	14	4	18	172.5	9.6
3	13	3	16	171.9	10.7	16	2	18	89.4	5.0
4	7	1	8	47.1	5.9	12	1	13	76.4	5.9
5	15	4	19	90.4	4.8	15	4	19	121.1	6.4
6	9	1	10	89.1	8.9	17	1	18	119.4	6.6
7	12	5	17	152.7	9.0	25	3	28	139.3	5.0
8	17	6	23	115.7	5.0	18	6	24	138.7	5.8
9	39	8	47	250.5	5.3	32	8	40	197.9	4.9
10	16	2	18	76.4	4.2	14	3	17	116.4	6.8
11	32	7	39	178.1	4.6	28	8	36	111.6	3.1
12	13	6	19	92.9	4.9	13	6	19	116.7	6.1
13	13	3	16	114.4	7.2	10	3	13	85.9	6.6
14	11	6	17	102.6	6.0	17	6	23	128.0	5.6
15	14	2	16	101	6.3	15	2	17	131.9	7.8
16	8	6	14	91.3	6.5	11	1	12	103.5	8.6
**Total**	**251**	**66**	**317**	**1911.9**	**101.9**	**272**	**59**	**331**	**1948.9**	**100.0**
Mean	13.5	4	17.5	101.8	6.0	15	3	18	118.1	6.2
Min	7	1	8	47.1	4.2	10	1	12	76.4	3.1
Max	39	8	47	250.5	10.7	32	8	40	197.9	9.6

*IP1 = individual markers (parent 1 specific); IP2 = individual markers (parent 2 specific); CM = markers common to both parents; TM = total number of markers for linkage group; cM = centiMorgan; AMD = Average marker density*.

**Table 4:** Characteristics of genetic linkage groups of mapping population DA41^1^.

Linkage group	DA41 Integrated Map (ARK86D×ML161P)						
	IM1	IM2	CM	TM	AP	cM	AMD
1	17	9	1	27	7	101.6	3.8
2	15	12	5	32	5	167.8	5.2
3	13	12	3	28	6	169.1	6.0
4	7	7	1	15	6	77.5	5.2
5	15	7	4	26	12	114.4	4.4
6	9	12	1	22	6	129.9	5.9
7	12	20	5	37	8	171.1	4.6
8	17	6	6	29	18	126.9	4.4
9	39	10	8	57	30	223.7	3.9
10	16	10	3	29	6	176.2	6.1
11	32	9	8	49	26	144.1	2.9
12	13	10	6	29	9	122.5	4.2
13	13	5	3	21	8	100.3	4.8
14	11	13	7	31	9	145.2	4.7
15	14	7	2	23	10	119.9	5.2
16	8	10	6	24	2	157.3	6.6
**Total**	**251**	**159**	**69**	**479**	**168**	**2247.5**	**77.9**
Mean	14	10	4.5	29	8	137	4.7
Min	7	5	1	15	2	77.5	2.9
Max	39	20	8	57	30	223.7	6.6

*IP1 = individual markers (parent 1 specific); IP2 = individual markers (parent 2 specific); CM = markers common to both parents; TM = total number of markers for linkage group; AP = number of anchor points; cM = centiMorgan; AMD = Average marker density*.

The ARK86D linkage map comprised 317 marker loci (236 SSRs, 75 AFLPs and 5 PCR– RFLPs) distributed on 16 linkage groups with 8–47 markers per group. The male parent ML161P linkage map had 331 marker loci (260 SSRs, 67 AFLPs and 4 PCR–RFLPs) distributed at 12–40 markers each in 16 linkage groups. The first map spanned 1,912 cM with an average map density of 6.0 cM while the latter map 1949 cM with a similar average density of 6.2 cM. For comparison, the distance of the dura DA10D map was 1,528 cM and the tenera parent LM2T estimated at 1597 cM [5]. Moretzsohn *et al.* reported parental genetic distances of 1,685 cM and 1,561 cM on a partial RAPD genetic map of their *tenera*×*pisifera* cross [2].

The 168 anchor points, based on codominant SSR and PCR–RFLPs marker alleles, allowed the determination of homologous groups for both maps and derivation of an integrated map. The latter was 2,247.5 cM long and included 479 marker loci (331 SSRs, 142 AFLPs and 6 PCR–RFLPs) at an average 29 markers and a range of 15–57 markers per linkage group, and an average map density of 4.7 cM. The linkage group lengths ranged from 77.5 cM to 223.7 cM, with an average 137 cM (Figures 1, 2, 3 and 4). For comparison, the *E. guineensis* integrated map based on 944 markers (255 SSRs, 688 AFLPs, allele *Sh*) was estimated to be 1,743 cM long [5] while a genetic distance of 1,815 cM and an average interval of 7 cM between adjacent markers for their *E. oleifera*×*E. guineensis* interspecific cross was reported [15].

## Discussion

### Mapping population

The very reasons which make marker-assisted breeding imperative for oil palm, namely, its cross-pollinating nature, long generation interval and large size, also make it particularly difficult to find suitable mapping populations. Existing genetically-defined populations are usually crosses between parents of varying heterozygosities arising from small founder populations, sometimes down to a single palm, that have subsequently been subjected to selection on a limited set of traits and inbreeding or outcrossing. The large size of the palm severely limits the number available for mapping as usually only 48–120 are planted per cross, the breeder having to choose between number of crosses and size of each cross in breeding trials. Furthermore, the search for marker(s) for the shell gene has veered most mapping work to *tenera*×*tenera* [1], [11], *dura*×*tenera* [4], [5] and *tenera*×*pisifera* [2] crosses as mapping populations. Only more recent interest in QTLs is expanding the types of populations analysed to include crosses, such as FELDA DA41, which are genetically closer to commercial planting materials as well as having larger populations which segregate for the QTLs of interest. Of particular interest is the high oil content of the fruit bunches of this cross, 35.4% in an early trial and 32.3–35.4% in three subsequent trials (FELDA, unpublished data). For comparison, current commercial plantings have a bunch oil content of ∼26%. The high bunch oil of DA41 derives from more mesocarp and higher oil content in the mesocarp. Fruit bunch production was average but the higher bunch oil resulted in a **8–21%** higher oil yield than the trial average in the four trials mentioned above.

### Genetic Markers

SSR markers offer many advantages for marker-assisted selection and will be the markers of choice for the FELDA marker breeding programme. They form the backbone of the present map. Of the 162 test cross SSR markers, 37% were found to be heterozygous descending from ARK86D and 63% heterozygous from ML161P. This suggests the male parent to be more heterozygous possibly due to less inbreeding. The female parent of DA41 is a Deli dura descended from at least seven generations of selfing, or sib-mating, from a gene pool of four founder palms first brought to the Bogor Botanic gardens, Indonesia in 1848. The absence of rare and low-frequency alleles in their Deli *dura* population was reported [6], [8] while 36 more RFLP alleles were found in germplasm *dura* populations compared to the Deli duras [7]. There were only 41 EST-SSR alleles found in Deli *dura*, where Ao was 2.7, compared to germplasm *dura* populations (Ao of 2.8–3.9) [9]. The male parent descended, through fewer generations of sib mating, from ancestral palms from a wider gene pool at an early oil palm research station at Yangambi, Congo. This finding of lower heterozygosity in the Deli dura population has also been previously reported [5].

In this study, the markers were well distributed over all the 16 linkage groups. There were two long intervals of 26.9 cM in Group III and 25.6 in Group IX, 7 (Fig. 1) suggesting them to be more homozygous regions, or where recombinations are not uniformly distributed as assumed by mapping algorithms [5], [20]. There were no intervals longer than 25 cM in any of the other groups, which is promising in the search and tagging of QTLs. The core markers in this study were SSRs given their advantages and utility when the work is later extended to other breeding crosses. SSR markers from in-house development and those provided by MPOB as well as CIRAD markers from the public domain were used. This is the first time that the FELDA and MPOB markers are being reported on. The CIRAD SSR markers mapped, except for two linkage groups, into 14 linkage groups, similar to those reported [5]. This lack of complete congruence is not unexpected given the very different genetic backgrounds of the populations used – for example only 144 (56.5% of the 255) of the CIRAD SSR markers were successfully mapped in this study. When the AFLP markers were added to saturate the map, the addition did not seriously disturb the original order of relative distances. It was likewise reported with RFLP markers [18], although some reported substantial expansion [36], [37]. In our study, the relatively large mapping population, informativeness of the, codominant, SSR markers and rejection of markers with unexpected segregation ratios may be responsible for length conservation as also reported elsewhere [38].

In conclusion, using 571 SSR, AFLP and PCR–RFLP markers from public domain publications, provided by MPOB and developed in-house, we constructed the first integrated genetic linkage maps of a FELDA high-yielding commercial oil palm cross and its parental palms. The maps share many characteristics with other oil palm maps as well as exhibit features which may be unique to the mapping population. The markers were fairly well distributed across 16 linkage groups though slightly more were mapped in the more heterozygous pisifera parent.

This work is a first step towards application of DNA markers to augment FELDA's oil palm breeding programme, recognising the potential contribution of the technology in breeding long-lived, long generation interval, high economic value plants. Current work is progressing along three tracks, a) continued map saturation, b) map validation from closely-related to divergent populations, and c) mapping of QTLs for yield components.
